# Working towards precision medicine in developmental programming

**DOI:** 10.1038/s41390-021-01466-x

**Published:** 2021-03-22

**Authors:** Dino A. Giussani

**Affiliations:** 1 Department of Physiology, Development and Neuroscience, University of Cambridge, Cambridge, UK; 2 Cambridge BHF Centre of Research Excellence, Cambridge, UK

The gene–environment interaction before birth is just as, if not more, important as the gene–lifestyle interaction after birth in setting a risk of disease in later life through a process known as developmental programming.^1^ The best evidence in humans to support developmental programming comes from studies of obese women who have fallen pregnant before and after having bariatric surgery.^2^ These studies show that siblings born before the surgery have an increased risk of cardiometabolic disease compared to those born after. Therefore, such studies underscore that alterations in the environment at critical periods of intrauterine development even within the same womb can directly influence long-term cardiovascular health in offspring of the same family. Consequently, there has been an exponential growth of studies in this field of developmental programming aiming to identify underlying mechanisms and thereby treatment, most recently focussed on improving precision medicine by applying basic principles of personalized medicine to intrauterine therapy.^3^ Two relevant examples are provided by studies aiming to improve organelle-targeted therapy^4–6^ and by the growing awareness that the sex of the offspring and of its placenta ought to be included as biological variables into experimental design and analysis when studying vertebrate animals and humans.^7–9^

A cluster of recent studies has aimed to improve precision intrauterine medicine by employing mitochondria-targeted, rather than conventional, antioxidants to protect against cardiovascular dysfunction programmed in offspring by chronic fetal hypoxia, one of the most common complications in human pregnancy.^4–6^ An interesting discussion is brewing comparing mitochondria-targeted antioxidant therapy directly to the fetus, or treatment specifically to the placenta. Studies using the chicken embryo show that direct in ovo administration of the mitochondria-targeted antioxidant MitoQ prevented in vivo mitochondria-derived oxidative stress and protected against cardiac mitochondrial dysfunction, and impaired cardiac and endothelial function in the chronically hypoxic chicken embryo.^4^ Further, studies in sheep show that maternal systemic treatment with MitoQ crosses the placenta, increases MitoQ uptake into the fetal tissues, and prevents programmed hypertension in the adult offspring.^4^ Combined, therefore, studies in the chicken embryo and in the sheep show that mitochondria-targeted therapy to the hypoxic fetus prevents programmed cardiovascular disease in the adult offspring.^4^ Conversely, there have been recent studies in rats, which have developed a placenta-targeted treatment strategy using MitoQ encapsulated into nanoparticles (nMitoQ). The aim is to protect against placental oxidative stress while preventing passage of the prenatal therapy into the fetal circulation, thereby avoiding potential adverse off-target effects on the fetus.^6^ These studies have also reported improved placental function and protection against cardiovascular dysfunction in adult offspring of hypoxic pregnancy.^6^

Similarly, there has been a growing awareness in the field of developmental programming that not only the sex of the fetus but also of its placenta can influence susceptibility to adverse intrauterine conditions and strategies to combat complications during pregnancy. Generally, it is widely accepted that the male fetus is at greater risk of morbidity and mortality in human complicated pregnancy.^7^ Clifton and colleagues^8^ have reported that in human pregnancies complicated by asthma, changes in placental function occur in a sex-specific manner, resulting in differences in fetal development, which influence child health. While the male placenta adapts function to maintain fetal growth at any cost, the female placenta triggers adaptations to budget resources and slow fetal growth, harnessing energy to survive a secondary insult.^8^ Evans and Myatt^9^ have also reported that the male fetus of a lean woman has the greatest antioxidant activity, and that this protection is lost with pregnancy complicated by maternal obesity.

In this issue of *Pediatric Research*, Wilson et al.,^10^ in an elegant study, report sexual dimorphism in the gene expression of the brain in a model of fetal growth restriction by maternal nutrient restriction in the guinea pig. In a pregnancy complicated by fetal growth restriction, inflammatory marker messenger RNA (mRNA) expression in the fetal brain was significantly higher in females compared to males. This provides evidence to refute the belief that it is always the male progeny that is at increased risk during a complicated pregnancy. The authors suggest that differences in gene expression between males and females may confer a selective advantage or disadvantage during adverse intrauterine conditions.^10^ Their studies also show that treatment of the placenta with a nanoparticle known to increase insulin-like growth factor 1 expression can not only protect fetal growth by increasing nutrient transporter expression and placental angiogenesis in adverse pregnancy but that it can also affect mRNA expression in the brain of the offspring in a sex-specific manner. Of most interest, despite only directly affecting the placenta, nanoparticle treatment resulted in changes in fetal brain mRNA expression of the inflammatory markers *Tgfβ* and *Ctgf*, so that the expression was similar between male and female fetuses in the nutrient-restricted groups.^10^ Combined, therefore, the observations in the study of Wilson et al.^10^ provide a good example of working towards precision medicine in the field of developmental programming not only by using targeted therapy but also by doing so using an experimental design that includes the sex of the fetus and of its placenta as biological variables. Only by adopting such approaches will real advances be made to expedite translational discoveries and isolate optimal therapy to improve the health of pregnant women and their children.
